# Evolution of Therapeutics for Locally Advanced Upper Gastrointestinal Adenocarcinoma

**DOI:** 10.3390/cancers17081307

**Published:** 2025-04-12

**Authors:** Jenny J. Li, Jane E. Rogers, Rebecca E. Waters, Qiong Gan, Mariela Blum Murphy, Jaffer A. Ajani

**Affiliations:** 1 Department of Gastrointestinal Medical Oncology, University of Texas M. D. Anderson Cancer Center, 1515 Holcombe Blvd, Houston, TX 77030, USA; mblum1@mdanderson.org (M.B.M.); jajani@mdanderson.org (J.A.A.); 2 Department of Pharmacy Clinical Program, University of Texas M. D. Anderson Cancer Center, 1515 Holcombe Blvd, Houston, TX 77030, USA; jerogers@mdanderson.org; 3 Department of Anatomical Pathology, University of Texas M. D. Anderson Cancer Center, 1515 Holcombe Blvd, Houston, TX 77030, USA; rwaters@mdanderson.org (R.E.W.); qgan@mdanderson.org (Q.G.)

**Keywords:** esophageal cancer, gastric cancer, perioperative treatment, immunotherapy, targeted therapy

## Abstract

Upper gastrointestinal (GI) malignancies, including esophageal, gastroesophageal junction (GEJ), and gastric adenocarcinomas, remain a major global health concern, with poor overall survival and high recurrence rate despite aggressive treatment. Patients with very early tumors (cT1a) can benefit from endoscopic therapy. However, patients with locally advanced disease require multimodal therapies that may combine surgery, radiation, and systemic therapies. This review provides a comprehensive overview of recent advancements in the treatment of locally advanced upper GI adenocarcinomas. Surgical resection remains the cornerstone of curative treatment, with perioperative chemotherapy emerging as the standard of care. While preoperative chemoradiation has demonstrated some benefits in esophageal and GEJ cancers, recent data suggest a more limited role for radiation going forward. Immunotherapy has shown some promise in both the adjuvant and perioperative settings but has yet to establish definitive survival benefit. The integration of HER2-targeted therapies into treatment regimens for HER2-positive locally advanced gastroesophageal cancers has not yielded significant improvements, underscoring the need for more effective strategies. Ongoing research focuses on better predictive biomarkers, personalized treatment approaches, and potential organ preservation strategies for patients achieving a clinical complete response. Continued advancements in treatment modalities and precision medicine are critical to improving survival for patients with locally advanced upper GI adenocarcinomas.

## Simple Summary

Cancers of the upper gastrointestinal tract, such as the esophagus, gastroesophageal junction (GEJ), and stomach, are difficult to treat. Even with aggressive management, the survival rates are low, and the recurrence rates are high. The current best treatment approach involves combining chemotherapy before and after surgery. While chemoradiation was an option previously to treat localized esophageal and GEJ cancers, it was found inferior to perioperative chemotherapy. Immunotherapy and targeted therapies have shown some promise, but they have not yet been proven to improve overall survival. Research is ongoing to find better ways to assess which therapy is most appropriate in an individual patient and to develop strategies that could help spare surgery in patients whose tumor is highly sensitive to the chosen therapy.

## 1. Introduction

Upper gastrointestinal (GI) malignancies, including cancers of the esophagus, gastroesophageal junction (GEJ), and stomach, represent a significant global healthcare burden. When combined, they rank as the fourth most common cancer and second leading cause of cancer-related deaths worldwide [1]. At diagnosis, about half of the patients have potentially curable nonmetastatic disease, but the 5-year overall survival (OS) remains suboptimal at only 21.6% for esophageal cancer and 36.4% for gastric cancer [2,3]. Even with the most aggressive curative intent therapy, recurrence rate for locally advanced disease remains high at 36% [4].

Within the last few years, data has emerged to define the roles of various modalities of therapy including surgery, radiation, and systemic therapies. While progress has been made, the optimal treatment strategy for locally advanced (cT2-4a, cN+/−, cM0) esophageal (EAC), GEJ (GEJ AC), and gastric adenocarcinomas (GAC) remains a subject of active investigation. In this review, we aim to provide a comprehensive overview of the most recent advancements in the management of locally advanced upper GI adenocarcinomas, focusing on multimodal therapies, preoperative/adjuvant/perioperative treatment approaches, and the integration of immunotherapy and targeted therapies.

## 2. Endoscopic and Surgical Strategies

For patients with very early lesions that have only mucosal invasion (cT1a), endoscopic resection can be considered for curative treatment. Endoscopic mucosal resection (EMR) is the more common technique used in the West, whereas endoscopic submucosal dissection (ESD) is used more commonly in Asia/Japan, though in recent years it is being adopted more in the West as well [5]. Endoscopic resection can be combined with ablation to eradicate high-grade dysplasia and residual Barrett’s esophagus. If high-risk features are found on pathology, such as positive margins, presence of lymphovascular invasion, and poorly differentiated histology, then additional treatment such as surgery may be needed [6]. Previously, patients with submucosal tumor extension (cT1b) were thought to have higher risk for lymph node metastases, so endoscopic resection was not used [7]. However, recent data showed that with appropriate patient selection and more accurate staging via diagnostic mucosal resection, some patients with cT1b disease may be spared from esophagectomy by undergoing ESD [8,9,10].

Surgical resection is the mainstay of curative treatment for most patients with locally advanced upper GI adenocarcinomas. For EAC, the standard surgical approach is esophagectomy with lymphadenectomy, which can be performed using an open, minimally invasive (robotic or laparoscopic), or hybrid technique [11]. For GAC, the standard surgical approach is subtotal or total gastrectomy with D2 lymph node dissection (extensive removal of lymph nodes in stations 1–12a) [12,13]. The surgical approach for GEJ AC is based on the Siewert classification. Siewert type I is defined as tumors with an epicenter 1 to 5 cm above the GEJ, Siewert type II is defined as tumors with an epicenter 1 cm above to 2 cm below the GEJ, and Siewert type III is defined as tumors with an epicenter 2 to 5 cm below the GEJ [14]. Siewert types I and II tumors are managed as esophageal cancers, while Siewert type III tumors are managed as gastric cancers. The goal of surgeries is to obtain negative margins (R0) and adequate lymph node dissection.

While surgery is an important component of curative-intent therapy, resection alone does not achieve the best outcomes for patients with locally advanced disease. Preoperative and perioperative therapies have both been shown to improve overall survival compared to surgery alone [15,16,17]. Because multimodality therapy is necessary to achieve the best outcomes, cases of locally advanced EAC, GEJ AC, and GAC should be reviewed at multidisciplinary tumor board. While most biomarkers used in the metastatic setting (such as programmed death-ligand 1 [PD-L1], claudin 18.2, and human epidermal growth factor receptor 2 [HER2]) do not currently impact treatment decisions, microsatellite instability (MSI) status should be assessed in all newly diagnosed cases > cT1a to determine the optimal treatment regimen.

## 3. Radiation

In the context of curative management, radiation is typically administered concurrently with chemotherapy to achieve improved tumor control and reduce risk of locoregional recurrence. Chemoradiation (CRT) has been studied in both the preoperative and adjuvant settings (Table 1). For treatment of locally advanced EAC and GEJ AC, preoperative CRT was once considered comparable to perioperative chemotherapy. However, recent data from the ESOPEC and TOPGEAR trials have brought the role of preoperative CRT into question [18,19]. Moving forward, CRT may be reserved for more limited clinical indications, such as definitive treatment for patients who are not candidates for surgery, or as a salvage option for those who do not respond adequately to chemotherapy.

### 3.1. Preoperative Chemoradiation

The role for preoperative CRT in locally advanced EAC and GEJ AC was established by the CROSS trial [15]. This phase 3 trial randomized patients to surgery alone (*n* = 188) or CRT followed by surgery (*n* = 178). CRT + surgery resulted in a higher R0 resection rate (92% vs. 69%, *p* < 0.001), improved median OS (49.4 months vs. 24 months, *p* = 0.003), and better disease-free survival (DFS) (not reached vs. 24.2 months, *p* < 0.001). These results established preoperative CRT as one of the standard-of-care treatment approaches for locally advanced esophageal and GEJ cancers, and it has remained so over the last decade until recent results from the ESOPEC trial.

For locally advanced GAC, the role for preoperative CRT was explored in the TOPGEAR trial [19]. TOPGEAR was an international phase 3 trial that randomized patients with resectable GAC to preoperative CRT + perioperative chemotherapy (*n* = 286) or perioperative chemotherapy alone (*n* = 288). While a higher percentage of patients achieved pathologic complete response (pCR) in the preoperative CRT group (17% vs. 8%), this did not translate to a significant difference in OS between the two groups. The median OS was 46 months in the preoperative CRT + perioperative chemotherapy group and 49 months in the perioperative chemotherapy group (hazard ratio 1.05). These results suggest that preoperative CRT provides no additional benefit over chemotherapy alone and that preoperative radiation has a very limited role in the management of locally advanced GAC.

### 3.2. Adjuvant Chemoradiation

The ARTIST, ARTIST 2, CRITICS, and MacDonald trials evaluated the role of adjuvant CRT in resected GAC. Both the ARTIST and ARTIST 2 trials compared postoperative chemotherapy versus CRT in GAC patients who underwent complete resection with D2 lymph node dissection [20,21]. Results from both trials showed that CRT did not significantly reduce the recurrence rate compared with chemotherapy alone. Likewise, the CRITICS trial, a randomized phase 3 study comparing perioperative chemotherapy (*n* = 393) to preoperative chemotherapy + postoperative CRT (*n* = 395), found no improvement in OS with postoperative CRT (37 months vs. 43 months, hazard ratio 1.01, *p* = 0.90) [22]. The only trial to demonstrate survival benefit for CRT in the adjuvant setting was the MacDonald trial, which was a phase 3 trial that randomly assigned patients with GAC/GEJ AC to receive either surgery + adjuvant CRT (*n* = 281) or surgery alone (*n* = 275) [23]. The addition of adjuvant CRT significantly prolonged the median OS compared with surgery alone (36 months vs. 27 months, *p* = 0.005, hazard ratio 1.52). Notably, only 10% of patients on this trial underwent D2 lymph node dissection, whereas 90% of patients had less than a D2 dissection. These results indicate the general lack of benefit of adjuvant CRT in patients who undergo adequate surgical resection, and adjuvant CRT only has a role in patients who undergo suboptimal surgeries.

## 4. Chemotherapy

### 4.1. Perioperative Chemotherapy

The MAGIC trial was the first landmark study to establish the perioperative approach for the management of locally advanced upper gastrointestinal adenocarcinomas (Table 2) [16]. Patients with resectable EAC, GEJ AC, and GAC were randomized to perioperative epirubicin + cisplatin + fluorouracil (ECF) x 6 cycles (*n* = 250) versus surgery alone (*n* = 253). In the perioperative arm, patients were treated with ECF three cycles preoperatively and three cycles postoperatively. Perioperative chemotherapy improved 5-year OS (36% vs. 23%, *p* = 0.009) as well as progression free survival (PFS) (hazard ratio for progression 0.66, *p* < 0.001). This trial demonstrated that perioperative chemotherapy was superior to surgery alone and is a valid treatment approach for patients with locally advanced disease.

FLOT4 was a phase 2/3 trial that compared the MAGIC regimen (*n* = 360) with the perioperative fluorouracil + docetaxel + oxaliplatin (FLOT) regimen (*n* = 356) [24]. In the FLOT arm, patients were treated with four cycles of FLOT preoperatively and four cycles of FLOT postoperatively. The FLOT arm significantly improved the median OS (50 months vs. 35 months, *p* = 0.012) and median DFS (30 months vs. 18 months, *p* = 0.0036) when compared with the MAGIC arm. These results established perioperative FLOT as a new standard-of-care regimen for locally advanced GAC and GEJ AC.

### 4.2. Adjuvant Chemotherapy

Because perioperative chemotherapy is the current standard of care treatment approach in the West, most patients with locally advanced disease should receive preoperative chemotherapy. However, there are cases where patients undergo upfront surgery (such as patients with gastric outlet obstruction necessitating urgent surgery or patients who were understaged prior to upfront surgery), and adjuvant chemotherapy should be considered. In contrast, the management of gastroesophageal cancers in Asia differs from Western approaches, and upfront surgery remains the preferred strategy for treatment of locally advanced disease in many practices in Asia (Japan/Korea/China/Taiwan); therefore, adjuvant therapy is still widely utilized, and many studies of adjuvant chemotherapy have been conducted in Asia. Notably, because of the differences in practice, outcomes from Western studies should not be directly compared with those from Asian studies.

The role of adjuvant chemotherapy was explored in the ACTS-GC, JACCRO-GC07, and CLASSIC trials. ACTS-GC was a randomized phase 3 trial where patients with locally advanced GAC were treated with surgery + adjuvant S-1 (an oral fluoropyrimidine) (*n* = 529) or surgery alone (*n* = 530) [25]. Adjuvant S1 significantly improved the 3-year OS compared with surgery alone (80.1% vs. 70.1%, hazard ratio 0.68, *p* = 0.003). The JACCRO-GC07 trial was another phase 3 study in which patients with resected locally advanced GAC were randomized to receive either adjuvant S1 + docetaxel (*n* = 453) or adjuvant S-1 alone (*n* = 459) [26]. The addition of docetaxel improved the 3-year relapse-free survival (67.7% vs. 57.4%, *p* = 0.0008) and OS (77.7% vs. 71.2%, *p* = 0.0076) over S-1 alone. The CLASSIC trial was a randomized phase 3 study that assessed capecitabine + oxaliplatin for 6 months postsurgery (*n* = 520) versus surgery alone (*n* = 515) [27]. Adjuvant chemotherapy significantly increased the 3-year DFS compared with surgery only (74% vs. 59%, *p* < 0.0001). Notably, all three trials required patients to undergo curative-intent D2 gastrectomy. Together, these trials support the use of adjuvant chemotherapy in patients who did not receive preoperative chemotherapy after undergoing D2 gastrectomy.

## 5. Preoperative Versus Perioperative Approach

Following the publication of results from the CROSS and MAGIC/FLOT4 trials, there was ongoing debate regarding the superiority of perioperative chemotherapy versus preoperative CRT. The NEO-AEGIS study was a randomized phase 3 trial that attempted to answer this question by enrolling patients with locally advanced EAC/GEJ AC to receive perioperative chemotherapy (MAGIC/FLOT; *n* = 184) versus trimodality therapy (CROSS; *n* = 178) [28]. It is important to note that during the course of this trial, the standard of care perioperative chemotherapy regimen changed from ECF to FLOT, but only 15% of the perioperative arm received FLOT. Based on the data that were collected, the OS was similar (median OS 48.0 months vs. 49.2 months; 3-year OS 55% vs. 57%, *p* = 0.82) between the perioperative and trimodality groups, but other outcomes (i.e., nodal downstaging, R0 resection, pathological complete response) favored the CROSS regimen. When these results were published, the consensus was that there was equipoise between the two treatment approaches.

However, the long-awaited ESOPEC results were finally published in January 2025 and more definitively addressed the question of FLOT vs. CROSS [18]. ESOPEC randomized patients with locally advanced EAC/GEJ AC to receive perioperative FLOT (*n* = 221) versus preoperative CRT (*n* = 217). The 5-year OS (50.6% vs. 38.7%), median OS (66 months vs. 37 months), PFS (51.6% vs. 35.0%), and pCR rate (16.7% vs. 10.1%) were all significantly higher in the FLOT group than in the CROSS group. These results established perioperative FLOT as the preferred treatment approach in patients with resectable locally advanced upper GI adenocarcinoma. A few caveats must be taken into consideration when interpreting these results, including the absence of adjuvant nivolumab in the preoperative CRT group, the lower-than-expected treatment completion rate in the preoperative CRT group (67.7% in ESOPEC vs. 91% in CROSS), and the lower radiation dose used in ESOPEC (41.4 Gy) compared with the North American standard (50.4 Gy). Despite these caveats, the OS data were compelling, and perioperative FLOT has been adopted as the standard-of-care treatment regimen for locally advanced resectable EAC, GEJ AC, and GAC.

The outcomes for patients with locally advanced disease undergoing curative-intent therapy are sobering, as the 5-year OS with perioperative FLOT is still only 50.6%. Efforts are ongoing to identify strategies for enhancing the effectiveness of FLOT. One such strategy draws from the rectal cancer treatment paradigm, where patients receive total neoadjuvant therapy (TNT), combining both systemic chemotherapy and CRT prior to surgery. The CALGB 80803 trial adopted this strategy and used positron emission tomography (PET) scan responses to tailor therapy [29]. In this phase 2 trial, patients with locally advanced EAC/GEJ AC were randomized to undergo induction chemotherapy with fluorouracil + oxaliplatin (FOLFOX) versus carboplatin/paclitaxel. Repeat PET after induction determined whether patients received CRT with the same chemotherapy (PET responders, defined as ≥ 35% decrease in the standardized uptake value [SUV]) or crossed over to the alternative chemotherapy for CRT (PET non-responders, defined as <35% decrease in SUV). However, the 5-year OS in the best responders was still only 53%. Additionally, a few other trials (including TOPGEAR) also showed no improvement in survival when combining chemotherapy and preoperative CRT [19,30]. Thus, there is a need for better strategies to meaningfully improve patient outcomes, and many studies are underway to explore the role of immunotherapy and targeted therapy in the curative setting.

## 6. Immunotherapy

Immunotherapy (IO) has transformed the treatment landscape for advanced/metastatic gastroesophageal cancers. However, to date, no study has demonstrated an OS benefit from adding IO to chemotherapy or CRT in patients with locally advanced adenocarcinomas. Below, we review the results from recent trials for preoperative, adjuvant, and perioperative immunotherapy, as well as data specific to the microsatellite instability-high/deficient mismatch repair (MSI-H/dMMR) population (Table 3).

### 6.1. Preoperative Immunotherapy

The PERFECT trial was a phase 2 feasibility trial for patients with resectable EAC/GEJ AC who were treated with atezolizumab + preoperative CRT (CROSS regimen) [31]. Forty patients were enrolled, and the pCR rate was 25% (10/40). When compared with historic controls from the Netherlands Cancer Registry after propensity score matching, there was no difference in pCR rate or median OS. Similarly, the ECOG-ACRIN EA2174 trial, which was a phase 2/3 study of perioperative nivolumab and ipilimumab in patients with locoregional EAC and GEJ AC, showed that the addition of nivolumab to preoperative CRT (CROSS regimen) did not improve the pCR rate (24.8% vs. 21.0%, *p* = 0.27) [32].

### 6.2. Adjuvant Immunotherapy

CheckMate 577, a phase 3 randomized placebo-controlled trial, assigned esophageal/GEJ cancer patients who underwent preoperative CRT followed by R0 resection and had residual pathological disease to receive nivolumab for 1 year (*n* = 532) vs. placebo for 1 year (*n* = 262). [33]. Adjuvant nivolumab significantly improved the median DFS (22.4 months vs. 11 months, *p* < 0.001). As a result, adjuvant nivolumab became the standard of care for patients undergoing trimodality therapy, though OS data are not yet available.

In contrast to CheckMate 577, the ATTRACTION-5 and VESTIGE trials did not show any benefit to adjuvant immunotherapy. ATTRACTION-5, a phase 3 randomized placebo-controlled trial, enrolled patients with locally advanced GAC/GEJ AC after D2 gastrectomy and randomized them to receive adjuvant nivolumab + chemotherapy (*n* = 377) versus placebo + chemotherapy (*n* = 378) [34]. There was no difference in the 3-year relapse-free survival between the nivolumab group and the placebo group (68.4% vs. 65.3%, *p* = 0.44). VESTIGE, a phase 2 randomized trial, enrolled patients with high risk EAC, GEJ AC, and GAC who underwent preoperative chemotherapy and surgical resection [35]. Patients were randomized to either adjuvant chemotherapy or adjuvant nivolumab + ipilimumab. Interim analysis showed that nivolumab + ipilimumab did not improve median DFS over chemotherapy (11.4 months vs. 20.8 months, *p* = 0.99) and the trial was stopped because of futility.

### 6.3. Perioperative Immunotherapy

As perioperative FLOT is currently considered the gold standard treatment for resectable locally advanced upper GI adenocarcinomas, there is significant interest in investigating the potential of combining perioperative chemotherapy with IO. KEYNOTE-585, a phase 3 randomized placebo-controlled trial, assessed perioperative chemotherapy + pembrolizumab versus perioperative chemotherapy + placebo in patients with resectable locally advanced GAC/GEJ AC [36]. Because the standard chemotherapy regimen changed during the course of the trial, there were two cohorts (main cohort: cisplatin + capecitabine/fluorouracil, *n* = 804; FLOT cohort, *n* = 203). In the main cohort, pembrolizumab significantly increased the pCR rate when compared with placebo (12.9% vs. 2.0%, *p* < 0.00001), but the differences in median event-free survival (44.4 months vs. 25.3 months, *p* = 0.0198) and median OS (60.7 months vs. 58.0 months, *p* = 0.174) did not reach statistical significance.

In parallel, the MATTERNHORN trial is a phase 3 randomized trial that evaluated perioperative FLOT + durvalumab versus FLOT + placebo in resectable GAC/GEJ AC patients [37]. Durvalumab significantly increased the pCR rate (19% vs. 7%, *p* < 0.00001), but OS data are still pending. Similarly, DANTE is a phase 2/3 randomized trial that assessed perioperative FLOT + atezolizumab versus FLOT + placebo [38]. Results from the phase 2 portion showed higher histopathologic complete regression rates for the atezolizumab group than the placebo group (24% vs. 15%, *p* = 0.032), but we still await the survival data. It is important to acknowledge that pCR rate is not always a surrogate for survival, and whether the pCR improvements seen in these trials will translate to survival benefit is to be seen.

### 6.4. Immunotherapy for MSI-H/dMMR Patients

Patients with MSI-H/dMMR disease represent a distinct subgroup in which IO can be used without the need for chemotherapy or CRT for the treatment of locally advanced disease. NEONIPIGA was a phase 2 trial that evaluated neoadjuvant nivolumab + ipilimumab and adjuvant nivolumab in localized MSI-H/dMMR GAC/GEJ AC (*n* = 32), and INFINITY was a phase 2 trial that assessed neoadjuvant tremelimumab + durvalumab in resectable GAC/GEJ AC (*n* = 15) [39,40]. While the sample sizes were small, both studies showed remarkable pCR rates of 58.6% (NEONIPIGA) and 60% (INFINITY). Larger confirmatory phase 3 trials are still pending, but these results have already led to an update in the National Comprehensive Cancer Network (NCCN) guidelines for neoadjuvant or perioperative immunotherapy. Because treatment decisions can be impacted, MSI/MMR status should be established in all newly diagnosed cases > cT1a to determine the best treatment approach.

## 7. Targeted Therapy

Biomarkers such as MSI, PD-L1, HER2, and claudin 18.2 are routinely assessed in patients with advanced/metastatic gastroesophageal cancers to guide therapeutic decisions. However, aside from MSI, these biomarkers currently do not significantly influence treatment decisions for locally advanced disease. HER2-targeted therapies have been well-established in the metastatic setting over the past decade, and more recently, therapies targeting claudin 18.2 have also demonstrated survival benefits. Because of this, targeted therapies are actively being investigated in the context of locally advanced disease, with HER2 being the most extensively studied. Despite numerous trials aimed at advancing the use of HER2-targeted therapies in the locally advanced setting, none have yielded positive results to date (Table 4). Factors such as alterations in the tumor microenvironment, the development of compensatory pathways, and tumor heterogeneity may all contribute to resistance to HER2-targeted treatments. There is a clear need for the development of more effective strategies to target HER2 and other alterations for more personalized treatment options.

### 7.1. Preoperative Targeted Therapy

RTOG 1010, a randomized phase 3 trial, enrolled patients with HER2-positive locally advanced EAC to receive preoperative CRT with (*n* = 102) or without trastuzumab (*n* = 101) [41]. Median DFS was 19.6 months in the CRT + trastuzumab group versus 14.2 months in the CRT group (*p* = 0.97). The addition of trastuzumab did not improve outcomes for patients undergoing preoperative CRT.

### 7.2. Perioperative Targeted Therapy

HER-FLOT was an exploratory phase 2 trial of perioperative FLOT + trastuzumab in patients with HER2-positive locally advanced GAC/GEJ AC [42]. Fifty-six patients were enrolled, and the pCR rate was 21.4%. PETRARCA, a randomized phase 2/3 trial for patients with HER2-positive resectable GAC/GEJ AC, assigned patients to receive either perioperative FLOT alone (*n* = 41) or perioperative FLOT + trastuzumab + pertuzumab (*n* = 40) [43]. The addition of trastuzumab + pertuzumab significantly increased the pCR rate compared with the FLOT alone (35% vs. 12%, *p* = 0.02). However, this trial was prematurely closed prior to transition to phase 3 because of the negative results and lack of survival benefit from the JACOB study (chemotherapy + trastuzumab + pertuzumab for metastatic HER2-positive GAC/GEJ AC). Additionally, the regimen of FLOT + trastuzumab + pertuzumab was also associated with considerable grade ≥3 diarrhea (41%), so it was difficult to tolerate. Finally, results from the INNOVATION trial were presented at the 2025 American Society of Clinical Oncology (ASCO) Gastrointestinal Cancers Symposium. INNOVATION, a randomized phase 2 trial, assigned patients with HER2-positive GAC/GEJ AC to receive perioperative chemotherapy alone (*n* = 35) or perioperative chemotherapy + trastuzumab (*n* = 67) or perioperative chemotherapy + trastuzumab + pertuzumab (*n* = 70) [44]. Cisplatin + capecitabine was initially used as the chemotherapy regimen, but it was later switched to FLOT after FLOT became the new standard of care regimen. The 5-year PFS was not significantly different between the groups (51.9% vs. 61.0% vs. 47.9%), and the 5-year OS also was not significantly different between the groups (60.5% vs. 67.5% vs. 62.6%). Because of these results, the standard-of-care treatment for HER2-positive locally advanced gastroesophageal adenocarcinomas remains FLOT.

## 8. Future Directions

Advancements in the treatment of locally advanced EAC, GEJ AC, and GAC have proven to be challenging. Despite multiple trials evaluating multimodality therapy and combinations of chemotherapy, immunotherapy, and targeted therapy, most of the trials have yielded negative results. Empiric trials have mostly failed to significantly improve patient outcomes. There is a scarcity of data on targeted therapies beyond those targeting HER2 (such as drugs targeting claudin 18.2) for locally advanced disease, and there is a clear need to find better predictive biomarkers and develop a more personalized approach for the management of locally advanced gastroesophageal adenocarcinomas.

In addition to establishing better targeted therapies, there is also increasing interest in the possibility of organ preservation and nonoperative management (“watchful waiting”) in patients with clinical complete response after preoperative therapy. This approach has been more commonly used in esophageal squamous cell carcinoma, which has a much higher pathologic complete response rate after preoperative therapy [15]. This approach is also being evaluated in MSI-H/dMMR patients (NCT06059495). In order to assess both biomarker-driven targeted therapies and the possibility of organ preservation, the American Association for Cancer Research (AACR) is developing a unique platform neoadjuvant biomarker-driven trial in patients with gastroesophageal adenocarcinoma, and more details will be available in the coming months.

Cellular therapies such as chimeric antigen receptor T-cell (CAR-T) therapy have emerged as a promising treatment modality in various cancers. While CAR-T therapy has been successful in hematologic malignancies, its applications have been limited in solid tumors [45]. For gastroesophageal cancers, CAR-T therapy remains experimental and is being studied in early-phase clinical trials. Common antigens targeted by CAR-T for gastroesophageal cancers include claudin 18.2 (NCT05539430, NCT04404595) and CEA (NCT06010862, NCT06821048). All ongoing trials are for advanced/metastatic disease, not for locally advanced disease with curative-intent treatment. Challenges for adapting CAR-T therapy for GI cancers include the heterogeneity of tumor target antigen expression, the complex immune microenvironment, and the risk of severe side effects such as cytokine release syndrome and neurotoxicity [46,47]. More data from ongoing trials are needed to fully understand the safety, efficacy, and long-term benefits of CAR-T therapy for curative treatment in these cancers.

Circulating tumor DNA (ctDNA) has shown potential as a prognostic biomarker and a tool for monitoring treatment response and detecting recurrence in patients with locally advanced gastroesophageal cancers. One exploratory study found that pretreatment ctDNA status more accurately stratified DFS than nodal status on PET [48]. Additionally, clearance of ctDNA after neoadjuvant treatment was associated with a favorable response to therapy [48]. Another observational study found that preoperative ctDNA detection was associated with high risk of recurrence [49]. Recent research has also explored the use of ctDNA as a strategy for watchful waiting in locally advanced gastroesophageal cancer, with clearance of ctDNA during neoadjuvant chemoradiation potentially indicating a lower risk of relapse [50]. However, while these findings are promising, they are very preliminary, and ctDNA is currently used only as a research tool. Larger prospective studies are needed to validate the application of ctDNA as a biomarker and to determine its potential role in guiding treatment decisions.

## 9. Conclusions

Over the last decade, significant advancements have been made in the treatment of locally advanced gastroesophageal adenocarcinoma. However, despite these developments, patient outcomes following curative-intent therapy remain suboptimal. Currently, perioperative chemotherapy is the standard of care for resectable locally advanced EAC, GEJ AC, and GAC regardless of biomarkers, with the exception of MSI-H/dMMR. Ongoing efforts to enhance treatment options include investigating the roles of immunotherapy and targeted therapies. As this is a rapidly evolving field, the standard of care is likely to shift in the near future. There is a critical need for more reliable predictive biomarkers and the development of novel targeted therapies to enable more personalized treatment strategies and, ultimately, improve patient outcomes.

## Figures and Tables

**Table 1:** Summary of major trials of chemoradiation in locally advanced gastroesophageal adenocarcinoma.

Trial Name	Phase	Disease Site	Setting	Intervention	Outcomes	Reference
CROSS	III	Esophagus, GEJ	Preoperative	Preoperative CRT + surgery vs. surgery alone	R0 resection 92% vs. 69%, *p* < 0.001 mOS 49.4 m vs. 24.0 m, HR 0.657, *p* = 0.003	[11]
TOPGEAR	III	GEJ, Gastric	Preoperative	Preoperative CRT + perioperative chemotherapy (ECF, FLOT) vs. perioperative chemotherapy alone	pCR 17% vs. 8% mOS 46 m vs. 49 m, HR 1.05	[14]
ARTIST	III	Gastric	Adjuvant	Adjuvant chemotherapy (XP) + CRT vs. adjuvant chemotherapy	3-year DFS 78.2% vs. 74.2%, *p* = 0.0862	[15]
ARTIST 2	III	Gastric	Adjuvant	Adjuvant S-1 vs. SOX vs. CRT (SOX + RT)	3-year DFS 64.8% vs. 74.3% vs. 72.8%, *p* = 0.879 (SOX vs. CRT)	[16]
CRITICS	III	GEJ, Gastric	Adjuvant	Perioperative chemotherapy vs. preoperative chemotherapy + postoperative CRT	mOS 43 m vs. 37 m, HR 1.01, *p* = 0.90	[17]
MacDonald	III	GEJ, Gastric	Adjuvant	Surgery alone vs. surgery + adjuvant CRT	mOS 27 vs. 36 m, HR 1.35, *p* = 0.005	[18]

Abbreviations: GEJ, gastroesophageal junction; CRT, chemoradiation; ECF, epirubicin + cisplatin + fluorouracil; FLOT, fluorouracil + docetaxel + oxaliplatin; XP, capecitabine + cisplatin; SOX, S-1 + oxaliplatin; mOS, median overall survival; pCR, pathologic complete response; DFS, disease-free survival; HR, hazard ratio.

**Table 2:** Summary of major trials of chemotherapy in locally advanced gastroesophageal adenocarcinoma.

Trial Name	Phase	Disease Site	Setting	Intervention	Outcomes	Reference
MAGIC	III	Esophagus, GEJ, Gastric	Perioperative	Perioperative ECF vs. surgery alone	5-year OS 36% vs. 23%, HR 0.75, *p* = 0.009	[12]
FLOT4-AIO	II/III	GEJ, Gastric	Perioperative	Perioperative FLOT vs. perioperative ECF/ECX	mOS 50 m vs. 35 m, HR 0.77, *p* = 0.012 mDFS 30 m vs. 18 m, HR 0.75, *p* = 0.0036	[19]
ACTS-GC	III	Gastric	Adjuvant	Surgery + adjuvant S-1 vs. surgery alone	3-year OS 80.1% vs. 70.1%, HR 0.68, *p* = 0.003	[20]
JACCRO-GC07	III	Gastric	Adjuvant	Adjuvant S-1 + docetaxel vs. adjuvant S-1 alone	3-year RFS 67.7% vs. 57.4%, HR 0.715, *p* = 0.0008 3-year OS 77.7% vs. 71.2%, HR 0.742, *p* = 0.0076	[21]
CLASSIC	III	Gastric	Adjuvant	Surgery + adjuvant CAPOX vs. surgery alone	3-year DFS 74% vs. 59%, HR 0.56, *p* < 0.0001	[22]

Abbreviations: GEJ, gastroesophageal junction; ECF, epirubicin + cisplatin + fluorouracil; ECX, epirubicin + cisplatin + capecitabine; FLOT, fluorouracil + docetaxel + oxaliplatin; CAPOX, capecitabine + oxaliplatin; mOS, median overall survival; mDFS, median disease-free survival; RFS, relapse-free survival; HR, hazard ratio.

**Table 3:** Summary of major trials of immunotherapy in locally advanced gastroesophageal adenocarcinoma.

Trial Name	Phase	Disease Site	Setting	Intervention	Outcomes	Reference
PERFECT	II	Esophagus	Neoadjuvant	Preoperative CRT + atezolizumab	pCR 25%	[27]
ECOG-ACRIN EA2174	II/III	Esophagus, GEJ	Neoadjuvant	Preoperative CRT + nivolumab vs. preoperative CRT alone	pCR 24.8% vs. 21.0%, *p* = 0.27	[28]
CHECKMATE-577	III	Esophagus, GEJ	Adjuvant	Adjuvant nivolumab vs. placebo	mDFS 22.4 m vs. 11.0 m, HR 0.69, *p* < 0.001	[29]
ATTRACTION-05	III	GEJ, Gastric	Adjuvant	Adjuvant chemotherapy (S-1, CAPOX) + nivolumab vs. adjuvant chemotherapy alone	3-year RFS 68.4% vs. 65.3%, HR 0.90, *p* = 0.44	[30]
VESTIGE	II	Esophagus, GEJ, Gastric	Adjuvant	Adjuvant nivolumab + ipilimumab vs. adjuvant chemotherapy	mDFS 11.4 m vs. 20.8 m, HR 1.55, *p* = 0.99	[31]
KEYNOTE-585	III	GEJ, Gastric	Perioperative	Perioperative chemotherapy (cisplatin + capecitabine/fluorouracil, FLOT) + pembrolizumab vs. perioperative chemotherapy alone	Main cohort: pCR 12.9% vs. 2.0%, *p* < 0.00001 mEFS 44.4 m vs. 25.3 m, HR 0.81, *p* = 0.0198 mOS 60.7 m vs. 58.0 m, HR 0.90, *p* = 0.174	[32]
MATTERHORN	III	GEJ, Gastric	Perioperative	FLOT + durvalumab vs. FLOT + placebo	pCR 19% vs. 7%, *p* < 0.00001	[33]
DANTE	II/III	GEJ, Gastric	Perioperative	FLOT + atezolizumab vs. FLOT + placebo	CRR 24% vs. 15%, *p* = 0.032	[34]
NEONIPIGA	II	GEJ, Gastric	Perioperative	Preoperative ipilimumab + nivolumab and postoperative nivolumab	pCR 58.6%	[35]
INFINITY	II	GEJ, Gastric	Neoadjuvant	Neoadjuvant tremelimumab + durvalumab	pCR 60%	[36]

Abbreviations: GEJ, gastroesophageal junction; CRT, chemoradiation; FLOT, fluorouracil + docetaxel + oxaliplatin; CAPOX, capecitabine + oxaliplatin; pCR, pathologic complete response; mOS, median overall survival; mDFS, median disease-free survival; RFS, relapse-free survival; mEFS, median event-free survival; CRR, complete regression rate; HR, hazard ratio.

**Table 4:** Summary of major trials of HER2-targeted therapy in locally advanced gastroesophageal adenocarcinoma.

Trial Name	Phase	Disease Site	Setting	Intervention	Outcomes	Reference
RTOG1010	III	Esophagus	Neoadjuvant	Preoperative CRT + trastuzumab vs. CRT alone	mDFS 19.6 m vs. 14.2 m, HR 0.99, *p* = 0.97	[37]
HER-FLOT	II	GEJ, Gastric	Perioperative	FLOT + trastuzumab	pCR 21.4%	[38]
PETRARCA	II	GEJ, Gastric	Perioperative	FLOT + trastuzumab + pertuzumab vs. FLOT alone	pCR 35% vs. 12%, *p* = 0.019	[39]
INNOVATION	II	GEJ, Gastric	Perioperative	Perioperative chemotherapy (cisplatin + capecitabine, FLOT) vs. chemotherapy + trastuzumab vs. chemotherapy + trastuzumab + pertuzumab	5-year PFS 51.9% vs. 61.0% vs. 47.9% 5-year OS 60.5% vs. 67.5% vs. 62.6%	[40]

Abbreviations: GEJ, gastroesophageal junction; CRT, chemoradiation; FLOT, fluorouracil + docetaxel + oxaliplatin; pCR, pathologic complete response; OS, overall survival; mDFS, median disease-free survival; PFS, progression-free survival; HR, hazard ratio.
